# Emerging Function of Fat Mass and Obesity-Associated Protein (Fto)

**DOI:** 10.1371/journal.pgen.1003223

**Published:** 2013-01-03

**Authors:** Timo D. Müller, Matthias H. Tschöp, Susanna Hofmann

**Affiliations:** 1 Institute for Diabetes and Obesity, Helmholtz Zentrum München, Munich, Germany; 2 Department of Medicine, Technische Universität München, Munich, Germany; 3 Institute of Experimental Genetics, Helmholtz Zentrum München, Munich, Germany; Stanford University School of Medicine, United States of America

Genome-wide association studies (GWAS) are a laborious but powerful tool to identify genetic risk factors associated with complex polygenic traits such as obesity [1], diabetes [2], or coronary artery disease [3]. The link between genetic variation in *FTO* and obesity was first described in a GWAS for type 2 diabetes [1] and was later independently confirmed in different populations all over the world. First described in 2007, genetic variation in *FTO* has since become one of the most solidly confirmed risk factor for polygenic obesity in humans; yet, information about how FTO affects metabolism is still scarce. Bioinformatic analyses suggest *FTO* codes for a Fe(II)- and 2-oxoglutarate–dependent nucleic acid demethylase [4], [5] that catalyzes demethylation of 3-methylthymine in single-stranded DNA [5]. However, how this proposed function of FTO is integrated into the complex network of energy metabolism control remains the object of intense scientific investigation.

Analyses of genetically engineered mouse models, in which the function of *Fto* is either eliminated [6]–[8] or enhanced [9], support a role of *Fto* in energy metabolism but are inconsistent as to whether *Fto* modulates caloric intake, energy expenditure, or both. In 2009, global lack of *Fto* was reported to result in a lean phenotype as a consequence of increased energy expenditure [7]. Similar results were reported by another study [8], but both studies share two pitfalls. First, interpreting energy expenditure differences can be challenging when body composition differences are also present (see below). Second, germline loss of *Fto* causes perinatal lethality and growth retardation, which may give rise to secondary effects that are unrelated to the mechanism by which variation in *FTO* affects human metabolism [7], [8]. Notably, homozygous mice carrying a loss-of-function missense mutation in the C-terminal domain of *Fto* (367F) show no signs of perinatal lethality or growth retardation; they are lean, exhibit normal food intake and, when normalized by body weight, show increased energy expenditure [6]. While these studies appear to point to a potential role of Fto in regulating energy expenditure rather than food intake, mice globally overexpressing *Fto* are obese, hyperphagic, and exhibit normal energy expenditure when corrected for body fat or lean tissue mass [9]. In line with these data, most human studies report that obesity-predisposing *FTO* alleles are associated with increased food intake, but not energy expenditure (Table 1) [10]–[13].

**Table 1:** Overview about the most relevant Fto studies analyzing food intake and energy expenditure in mice and humans.

Species	Individuals/Assessed Phenotype	SNP Analyzed	BW (Mouse) BMI (Human)	Energy Intake	EE	Reference
Mouse	Global germline Fto ko	n/a	↓	↑	↑	Fischer et al., (2009) Nature 458: 894–898
Mouse	Global germline Fto ko	n/a	↓	→↑	↑	Gao et al., (2010) PLOS ONE 5: e14005
Mouse	CNS-specific Fto ko	n/a	↓	→↑	↑	Gao et al., (2010) PLOS ONE 5: e14005
Mouse	Global LOF Fto missense mutation	n/a	↓	→	↑	Church et al., (2009) PLOS Genet 5: e1000599
Mouse	Global Fto overexpression	n/a	↑	↑	→	Church et al., (2010) Nat Genet 42: 1086–1092
Human	150 Scottish Caucasians	rs9939609	↑	↑	→	Speakman et al., (2008) Obesity (Silver Spring) 16: 1961–1965.
Human	151 German subjects	rs8050136	↑	↑	→	Haupt et al., (2009) Exp Clin Endocrinol Diabetes 117(4): 194–197
Human	97 Scottish children	rs9939609	↑	↑	→	Cecil et al., (2008) N Engl J Med 359(24): 2558–66.
Human	2,075 participants from the Look AHEAD (Action for Health in Diabetes) clinical trial	rs1421085, rs3751812, rs9922708	n/a	↑	n/a	McCaffery et al. (2012) Am J Clin Nutr 95(6): 1477–86
Human	711 Korean children	rs9939973, rs9939609	↑	↑	n/a	Lee et al., (2010) Clin Chim Acta 411(21–22): 1716–22.
Human	1978 European- and African-American youth	rs9939609	↑	→	n/a	Liu et al., (2010) BMC Med Genet 11: 57
Human	438 Healthy participants of the STRIP Study	rs9939609	↑	→	n/a	Hakanen et al., (2009) J Clin Endocrinol Metab 94(4): 1281–7
Human	234 obese and 323 controls from Copenhagen	rs9939609	↑	n/a	→	Berentzen et al., (2008) J Clin Endocrinol Metab 93(7): 2904–8
Human	908 individuals from the Quebec City metropolitan area	rs17817449	↑	n/a	→	Do et al., (2008) Diabetes 57: 1147–1150
Human	908 individuals from the Quebec City metropolitan area	rs1421085	↑	n/a	→	Do et al., (2008) Diabetes 57: 1147–1150

In summary, despite intense scientific discussion about whether Fto primarily affects energy expenditure or food intake [14], it remains unclear what role Fto plays in early development compared to adult life and which tissues and/or brain regions are involved in mediating the effects seen in the global *Fto* knock-out (ko) mice. An important step in solving these questions has now been taken by Roger Cox and colleagues. In the current issue of *PLOS Genetics*, McMurray et al. [15] report a series of elegant studies further elucidating the complexity of Fto with respect to how, when, and where it is most relevant for energy metabolism and shedding new light on the recently proposed role of Fto in protein metabolism [16].

In their manuscript, the authors recapitulate that germline loss of *Fto* leads to perinatal lethality, growth retardation, and a lower body weight that is accompanied by decreased body fat and lean tissue mass. However, in contrast to previous reports, the authors convincingly show that there is no difference in energy expenditure when the data are interpreted correctly, i.e., using a regression approach that takes into account potential confounding by differences in lean body mass. Several review articles have recently highlighted this issue [17], [18], and it is now clear that simply dividing raw energy expenditure results by lean body mass can lead to spurious conclusions. (This is illustrated quite nicely in Figure 3 of McMurray et al., where an apparent difference of energy expenditure per gram of lean body mass vanishes upon regression adjustment using ANCOVA.) Interestingly, total food intake was not changed in the germline *Fto* ko mice, whereas the ratio between CO_2_ production and O_2_ consumption (respiratory exchange ratio; RER) was decreased, suggesting that *Fto* ablation promotes protein and/or fat utilization.

To circumvent the challenge of perinatal lethality and growth retardation, McMurray et al. used tamoxifen-inducible ubiquitin-cre mice to delete *Fto* at the time of sexual maturity. These adult onset *Fto* ko mice showed no increased lethality or growth retardation but had a lower body weight accompanied by a decreased lean mass and, interestingly, an increased body fat mass. No changes were observed in energy expenditure or total food intake, but, similar to the germline *Fto* ko, RER decreased in adult onset Fto ko mice, an effect also noted in a recent human study [19].

As central nervous system (CNS)-specific *Fto* deletion was recently reported to recapitulate the phenotype of the germline *Fto* ko mice [8], McMurray et al. further used an adenoviral associated approach to specifically knock out *Fto* in the mediobasal hypothalamus (MBH). Interestingly, these adult onset hypothalamic *Fto* ko mice showed no change in total body weight or body composition compared to sham controls but displayed a slightly decreased body weight gain that was accompanied by decreased food intake without any change in energy expenditure or RER. Taken together, these data indicate that although perturbation of Fto signaling in the MBH can impact food intake, sites other than the hypothalamus may be more important for Fto's influence on body composition and energy homeostasis.

In summary, Cox and colleagues with their current publication have made several important contributions that allow for a better understanding and potentially improved targeting of Fto signaling in the control of energy homeostasis. The authors make a convincing case that Fto may not directly affect energy expenditure in mice, thereby also shedding some light on a complex methodological question. The authors furthermore show that adult, rather than perinatal, loss of Fto is well tolerated, enabling the analysis of Fto effects without the confounding factors associated with perinatal lethality or growth retardation. The authors also show that lack of Fto in the hypothalamus explains only a small part of the phenotype observed in the global Fto ko mice, indicating that Fto promotes its biological effects through other, non-hypothalamic pathways. Finally, the observation that RER is decreased in the germline and adult onset Fto ko mice points to a role of Fto in regulating peripheral metabolism and substrate utilization. The current paper by Cox and colleagues along with the other studies reviewed here highlight both the considerable challenges of, and the need for, careful and often time-consuming functional studies before the value of GWAS candidate genes can be truly appreciated.
